# Different Diabetogenic Response to Moderate Doses of Streptozotocin in Pregnant Rats, and Its Long-Term Consequences in the Offspring

**DOI:** 10.1155/EDR.2003.107

**Published:** 2003

**Authors:** Iliana López-Soldado, Emilio Herrera

**Affiliations:** 1 Facultad de Ciencias Experimentales y de la Salud Universidad San Pablo–CEU Madrid Spain; 2 Universidad San Pablo–CEU Ctra. Boadilla del Monte km 5 300, E-28668 Boadilla del Monte Madrid Spain

## Abstract

Diabetes during pregnancy results in congenital malformations and long-term postnatal diseases. Experimental models are still needed to investigate the mechanism responsible for these alterations. Thus, by the administration of different doses of streptozotocin (STZ) (0, 25, 30, or 35 mg/kg body weight, intravenous) at the onset of pregnancy in rats, the present study sought an appropriate animal model for this pathology. At day 6 of pregnancy, plasma glucose was progressively higher with an increasing STZ dose, and in rats receiving the 35-mg dose, 2 subgroups were detected: some animals had plasma glucose levels above controls but below 200 mg/dL (mildly diabetic, MD), whereas others had levels above 400 mg/dL (severely diabetic, SD). At day 20 of pregnancy, the MD rats had normal glycemia, but after an oral glucose load (2 g/kg body weight), plasma glucose increased more and insulin increased less than in controls. The SD rats maintained their hyperglycemia and had a greatly impaired oral glucose tolerance. At day 20, fetuses of SD dams were fewer, weighed less, and had enhanced plasma glucose and triglycerides and decreased insulin, whereas those from MD dams did not differ from controls. At birth, newborns from MD dams had higher body weight, plasma insulin, and liver triglycerides as well as total body lipid concentrations than controls, and on day 21, remained macrosomic and showed higher plasma glucose and liver triglyceride concentrations. At 70 days of age, offspring of MD dams had impaired oral glucose tolerance but normal plasma insulin change in the case of females, whereas plasma insulin increased less in males. These alterations were manifest more in those offspring from dams that had > 50% macrosomic newborns than in those from dams that had < 50% macrosomic newborns. In conclusion, whereas our MD rats mimic the changes taking place in gestational diabetic women and show the long-term risk of macrosomia, the SD rats are more similar to uncontrolled diabetics. Thus these two rat models, obtained with moderate amounts of STZ, could be used to study the pathophysiological consequences of these different diabetic conditions.


*Experimental Diab. Res.*, 4:107–118, 2003
Copyright © Taylor and Francis Inc.
ISSN: 1543-8600 print / 1543-8619 online
DOI: 10.1080/15438600390220043

### Different Diabetogenic Response to Moderate Doses of Streptozotocin in Pregnant Rats, and Its Long-Term Consequences in the Offspring

**Iliana López-Soldado and Emilio Herrera**

*Facultad de Ciencias Experimentales y de la Salud, Universidad San Pablo–CEU, Madrid, Spain*

**Diabetes during pregnancy results in congenital malformations and long-term postnatal diseases. Experimental models are still needed to investigate the mechanism responsible for these alterations. Thus, by the administration of different doses of streptozotocin (STZ) (0, 25, 30, or 35 mg/kg body weight, intravenous) at the onset of pregnancy in rats, the present study sought an appropriate animal model for this pathology. At day 6 of pregnancy, plasma glucose was progressively higher with an increasing STZ dose, and in rats receiving the 35-mg dose, 2 subgroups were detected: some animals had plasma glucose levels above controls but below 200 mg/dL (mildly diabetic, MD), whereas others had levels above 400 mg/dL (severely diabetic, SD). At day 20 of pregnancy, the MD rats had normal glycemia, but after an oral glucose load (2 g/kg body weight), plasma glucose increased more and insulin increased less than in controls. The SD rats maintained their hyperglycemia and had a greatly impaired oral glucose tolerance. At day 20, fetuses of SD dams were fewer, weighed less, and had enhanced plasma glucose and triglycerides and decreased insulin, whereas those from MD dams did not differ from controls. At birth, newborns from MD dams had higher body weight, plasma insulin, and liver triglycerides as well as total body lipid concentrations than controls, and on day 21, remained macrosomic and showed higher plasma glucose and liver triglyceride concentrations. At 70 days of age, offspring of MD dams had impaired oral glucose tolerance but normal plasma insulin change in the case of females, whereas plasma insulin increased less in males. These alterations were manifest more in those offspring from dams that had >50% macrosomic newborns than in those from dams that had <50% macrosomic newborns. In conclusion, whereas our MD rats mimic the changes taking place in gestational diabetic women and show the long-term risk of macrosomia, the SD rats are more similar to uncontrolled diabetics. Thus these two rat models, obtained with moderate amounts of STZ, could be used to study the pathophysiological consequences of these different diabetic conditions.**

**Keywords** Macrosomia; Pregnancy; Rat; Streptozotocin Diabetes

Diabetes of the mother during pregnancy causes an abnormal intrauterine metabolic and hormonal milieu that results in congenital malformations and neonatal hypoglycemia [1, 2]. It also enhances the risk of short- and long-term postnatal disease, including macrosomia [3, 4], an increase in the frequency of insulin resistance, gestational diabetes [5, 6], and obesity [7–9]. Besides hyperglycemia, the multifactorial metabolic derangement resulting from maternal insulin deficiency seems to play an important role in those fetal disturbances [10]. Studies in humans that explore the responsible mechanism for these alterations are limited not only by ethical reasons but also by the multiplicity of uncontrolled variables that may modify the intrauterine environment and cause potential effects on congenital malformations, such as feeding behavior, socioeconomic factors, nutritional status, and genetic factors. Thus, there is a need for appropriate animal models. Models that use glucose

Received 12 May 2002; accepted 3 March 2003.
This study was supported by grants 0023/00 from the Dirección General de Investigación de la Comunidad de Madrid and 10/2000 from Universidad San Pablo–CEU, Spain. The technical assistance of Mrs. Milagros Morante and the editorial help of Mrs. Linda Hamalainen are greatly appreciated.
Address correspondence to Dr. Emilio Herrera, Universidad San Pablo–CEU, Ctra. Boadilla del Monte km 5,300, E-28668 Boadilla del Monte, Madrid, Spain. E-mail: eherrera@ceu.es infusions to produce maternal hyperglycemia do not duplicate maternal insulin deficiency [11, 12]. Because streptozotocin (STZ)-induced diabetes is now well characterized [13], this antibiotic agent has been widely used for inducing pancreatic $\beta$-cell degranulation and necrosis in pregnant rats. Whereas in some studies, STZ has been administered before the onset of pregnancy [14–20], in others, the drug is administered just at the onset [21–25], and even given on days 5 to 8 of gestation [26]. The first procedure, however, causes difficulties in obtaining successful mating, making it necessary to administer therapeutic insulin treatment during the mating period [14–17]. The last procedure is not free of secondary effects, because STZ is known to cross the placenta [27] and therefore may have direct effects on embryo development. The treatment with STZ on the day of onset of pregnancy is, nevertheless, the most convenient, because it does not affect the mating period and it certainly does not disturb the early stages of embryo development because of its very short half-life [28]. The role of STZ treatment in developing diabetes in the pregnant rats and its consequences in fetal or newborn body weight is highly variable, ranging from developing microsomia [21, 29–31], to no change [21, 32, 33] or to macrosomia [26, 34–36]. Whereas a positive correlation between maternal glycemia and fetal weight was found in mildly diabetic rats, a negative correlation between these two variables was found in severely diabetic rats [30]. Thus the severity of the diabetes attained determines the type of response. The difficulties of obtaining a mild-to-moderate hyperglycemia as a response to STZ treatment has forced researchers to look for experimental strategies such as the syngeneic islet transplant [37, 38] or insulin pump implantation after treatment with STZ [39], which, aside from their intrinsic difficulty, are far from mimicking the diabetic condition in humans. More recently, a model of gestational diabetes based on the offspring of uteroplacental insufficient pregnant rats has been described [40], showing that defects in glucose homeostasis in the offspring of diabetic pregnant rats lead to the development of diabetes later in life. In view of difficulties in obtaining an animal model of diabetic pregnancy, the present study aimed to define the dose of STZ to obtain an easy and reproducible animal model of mildly diabetic pregnancy, by administrating different doses of STZ at the onset of pregnancy in rats and studying them at different time points, and to study some of the short- and long-term pathophysiological consequences in the offspring.

### MATERIAL AND METHODS

Female Sprague-Dawley rats from our own colony, weighing 170 to 180 g, housed in a temperature-controlled room (21°C to 23°C) with 12-hour light-dark cycles and fed a standard nonpurified diet (B&K Universal, Barcelona, Spain) ad libitum, were mated. The day that spermatozoids appeared in vaginal smears (day 0 of pregnancy), they were intravenously treated with 25, 30, or 35 mg STZ (Sigma, St. Louis, MO, USA)/kg body weight, dissolved in 50 mM citrate buffer, pH 4.5. Control animals were run in parallel, and received the medium. The experimental protocol was approved by the Animal Research Committee of the University San Pablo–CEU in Madrid, Spain. On day 6 of pregnancy, animals were weighed and blood was collected from the tail into tubes containing $Na_2$-EDTA. On day 20 of pregnancy, some rats from controls and from those that were treated with the 35-mg STZ dose were killed by decapitation and blood from the neck wound was collected into tubes containing $Na_2$-EDTA. The two uterine horns were immediately excised and weighed with their contents to obtain the whole conceptus weight. This value was subtracted from the rat's total body weight to obtain the net maternal body weight. Blood from all the fetuses coming from the same dam was pooled and processed in parallel to that of the adults. In another set of rats from the same groups (e.g., controls and those treated with the 35-mg STZ dose), an oral glucose tolerance test (OGTT) was performed as follows: after collecting blood from the tail (0 time), the rats received an oral load of 2 g of glucose/kg body weight, and blood was collected at 7.5, 15, 22.5, 30, and 60 minutes. Rats showing a moderate diabetic condition (i.e., normal basal glucose but impaired OGTT) and controls were allowed to deliver, and the newborn pups immediately weighed thereafter. Litters were adjusted at random to 9 pups per dam and allowed to suckle. The day of birth all the remaining pups from each litter were decapitated and blood collected from the neck into tubes containing $Na_2$-EDTA. Livers were immediately excised and placed into liquid $N_2$ and kept at −80°C until processed for triglyceride (TG) analysis, as previously described [41]. Other newborn pups were directly placed into liquid $N_2$ and stored at −80°C until processed for lipid extraction [42] to determine total lipid content by weight of dried lipid extracts. On day 21 of lactation, pups were weaned. They were weighed and some of the pups from each litter were killed to collect blood as above. Livers were immediately excised and placed into liquid nitrogen and kept at −80°C until processed for lipid extraction [42]. TGs were quantified in aliquots of lipid extracts after image analysis and separation by 1-dimensional thin-layer chromatography (TLC) [43] using the G5-700 Bioimage TLC scanner of Bio-Rad (Hercules, CA, USA) as previously described [41]. The remaining pups were allow to progress until 70 days old, when they were subjected to an OGTT as above.

Plasma was immediately separated after blood collections by centrifugation at 3000 rpm for 20 minutes at 4°C, and was kept at −80°C until analysis for glucose, TGs, and immunoreactive insulin by using commercial kits (Boehringer-Mannheim,

**TABLE 1**
Plasma glucose and body weight at day 6 of gestation of control rats and rats that had received different doses of STZ (mg/kg body weight) at the onset of pregnancy

| | | | | STZ-35* | |
|---|---|---|---|---|---|
| | Controls | STZ-25 | STZ-30 | MD | SD |
| Blood glucose (mg/dL) | 126 ± 3[a] (14) | 141 ± 3[a] (12) | 160 ± 5[b] (15) | 169 ± 8[b] (17) | 424 ± 15[c] (5) |
| Body weight (g) | 221 ± 4[a] (14) | 219 ± 3[a] (12) | 216 ± 4[a] (15) | 216 ± 4[a] (17) | 201 ± 3[a] (5) |

*Note*. Values are means ± SEM. The number of observations is in parenthesis. Tukey's test was used to determine differences between groups after ANOVA. Different superscripts in a row indicate significance differences ($P < .05$).
*Those receiving the 35-mg/kg dose are differentiated into two groups: MD = mildly diabetic; SD = severely diabetic.

Germany; Menarini Diagnostics, Italy; and Mercodia AB, Sweden, respectively).

Results were expressed as means ± SEM. Values were analyzed by 1-way analysis of variance (ANOVA), using computer software (Systat Version 5.03, Wilkinson, Evanston, IL, USA). When treatment effects were significantly different ($P < .05$), specific means were tested by Tukey's test. Differences between the 2 groups were analyzed by Student's $t$ test.

### RESULTS

At day 6 of gestation, plasma glucose was progressively higher in rats that received the 25, 30, or 35 mg STZ dose/kg at the onset of gestation, although the difference with the controls was significant only in those receiving the 30-mg dose and over (Table 1). In rats that had received the 35-mg dose, 2 clearly differentiated subgroups could be detected: some animals showed a significant but moderate increase in plasma glucose (below the 200 mg/dL value), and this subgroup was named "mildly diabetic" (MD). In the others, plasma glucose levels were above 400 mg/dL and this group was named "severely diabetic" (SD). At this time of pregnancy, body weights did not differ between the groups (Table 1). As shown in Table 2, at day 20 of gestation, rats that had received the 35-mg STZ dose and were considered MD showed net maternal body weight (free of conceptus), plasma glucose, insulin, and TG levels, and fetal body weight that did not differ from those of controls. However, in comparison to both control and MD, rats that had received the same STZ dose (35 mg/kg) but were considered SD showed decreased net maternal body weight and fetal body weight, but increased plasma glucose and TG levels, as well as fetal plasma glucose. Whereas fetal plasma insulin appeared lower in this group than in MD rats, fetal plasma TGs were higher than controls (Table 2). No difference in either of these variables was found at day 20 of gestation in those rats that received the 25- or 30-mg STZ dose as compared to either controls, or rats receiving the 35-mg dose considered MD (data not shown).

At day 20 of gestation, some rat mothers from the MD, SD, and control groups were subjected to an OGTT. As shown in Figure 1, blood glucose levels were highly augmented in the

**TABLE 2**
Plasma glucose, insulin, and triglycerides, and body weight of mothers and fetus at day 20 of gestation of control rats and rats that had received 35 mg STZ/kg body weight at the onset of pregnancy

| | Controls | MD* | SD* |
|---|---|---|---|
| Maternal plasma glucose (mg/dL) | 89 ± 3[a] (10) | 83 ± 4[a] (13) | 188 ± 33[b] (9) |
| Maternal plasma triglycerides (mg/dL) | 416 ± 68[a] (10) | 384 ± 21[a] (13) | 1043 ± 83[b] (9) |
| Maternal plasma insulin ($\mu$U/mL) | 24.7 ± 3.8[a] (9) | 17.3 ± 2.9[a] (12) | 26.6 ± 4.3[a] (6) |
| Net maternal body weight (free of conceptus) (g) | 279 ± 4[a] (10) | 263 ± 3[a,b] (15) | 254 ± 10[b] (9) |
| Fetal plasma glucose (mg/dL) | 44 ± 3[a] (10) | 64 ± 11[a] (15) | 223 ± 35[b](9) |
| Fetal plasma triglycerides (mg/dL) | 69 ± 2[a] (10) | 72 ± 2[a,b] (15) | 79 ± 3[b](9) |
| Fetal plasma insulin ($\mu$U/mL) | 113 ± 10[a,b] (10) | 159 ± 13[b] (15) | 87 ± 17[a] (9) |
| Fetal body weight (g) | 4.70 ± 0.04[a] (8) | 4.62 ± 0.08[a] (11) | 3.94 ± 0.12[b] (8) |

*Note*. Values are means ± SEM. Fetal blood samples from each mother were pooled, and fetal weight values correspond to the means of all the fetuses from the same mother. The number of observations is in parenthesis. Tukey's test was used to determine differences between groups after ANOVA. Different superscripts in a row indicate significant differences ($P < .05$).
*Rats dosed with 35 mg STZ/kg body weight were separated into mildly diabetic (MD) and severely diabetic (SD) based on glycemia at day 6 of pregnancy.

A)

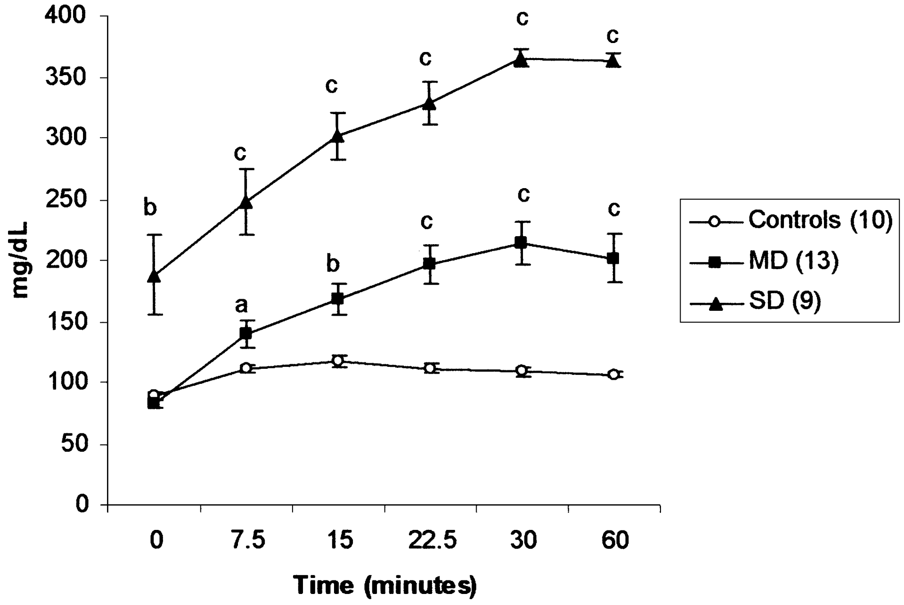

B)

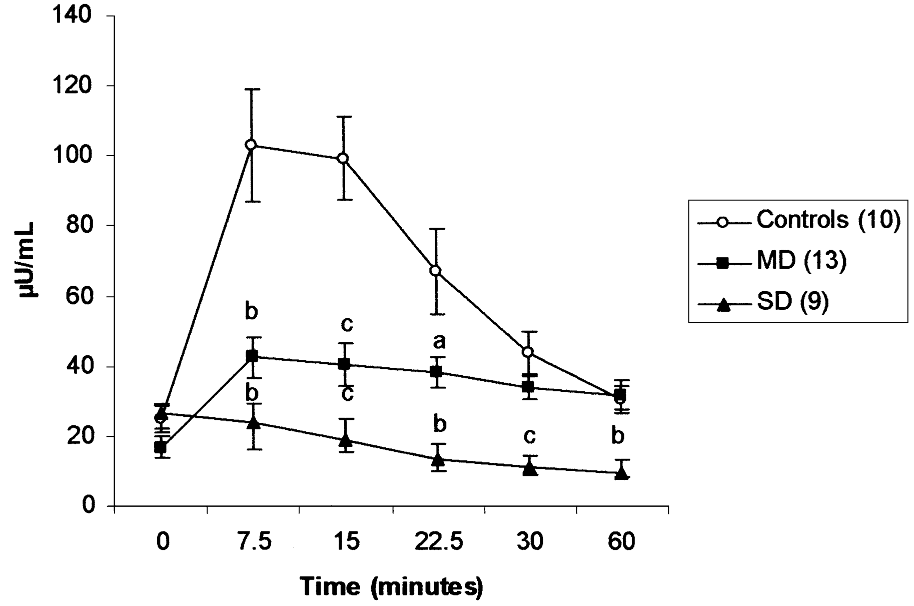

**FIGURE 1**

Plasma glucose (*A*) and insulin (*B*) levels at different times after an oral glucose load (2 g/kg body weight) at day 20 of pregnancy in mildly diabetic (MD) and severely diabetic (SD) rats that had received 35 mg/kg of streptozotocin at the onset of pregnancy, as compared to nontreated controls. Significant differences versus controls at each time point are shown by the letters: a, $P < .05$; b, $P < .01$; c, $P < .001$. The number of rats per group are shown in parenthesis.

SD rats as compared to controls at all time points studied. Although plasma insulin did not differ between the 2 groups at the 0- and 60-minute time point values at 7.5, 15, 22.5, and 30 minutes were significantly lower in the SD, which at no time showed any increase in plasma insulin as compared to basal levels (time 0). In the MD rats, although basal blood glucose levels did not differ from controls, values at 7.5, 15, 22.5, 30, and 60 minutes were significantly higher than in controls, and

**TABLE 3**
Body weight, plasma glucose and insulin, liver triglycerides, and total lipids in newborn (the day of birth) and 21-day-old pups of mildly diabetic (MD) and control rats

| | Newborns | | 21-day-old pups | |
|---|---|---|---|---|
| | Controls | MD | Controls | MD |
| Body weight (g) | 6.02 ± 0.04 (98) | 6.49 ± 0.06[c] (115) | 41.8 ± 0.4 (53) | 44.5 ± 0.5[c] (71) |
| Plasma glucose (mg/dL) | 86 ± 4 (10) | 103 ± 9 (8) | 158 ± 3 (13) | 169 ± 3[a] (11) |
| Plasma insulin ($\mu$U/mL) | 5.5 ± 1 (7) | 13.9 ± 3.6[a] (6) | 24.1 ± 1.4 (13) | 21.5 ± 1.9 (16) |
| Liver triglycerides (mg/g tissue) | 2.42 ± 0.09 (6) | 16.16 ± 6.58[a] (5) | 2.7 ± 0.3 (12) | 3.8 ± 0.2[b] (11) |
| Total lipids (mg/g) | 19.8 ± 2.45 (9) | 28.9 ± 2.1[a] (8) | ND | ND |

*Note*. Values are means ± SEM. $^aP < .05$; $^bP < .01$; $^cP < .001$ versus the corresponding controls. The number of observations is in parenthesis. ND, not determined.

plasma insulin levels were lower at 7.5, 15, and 22.5 minutes, with no difference between these 2 groups at 0, 30, or 60 minutes. In fact, contrasting with the SD rats, in the MD rats there was a significant increase in plasma insulin levels after the oral glucose load, although in the peak time, values were well below those of controls (Figure 1).

When MD and control rats were allowed to deliver, newborns weighed more and plasma insulin levels were higher in the first group. Blood glucose did not differ between the 2 groups and liver TG and total body lipid concentrations were higher in newborns from MD rats than in controls (Table 3). Some of the pups from these two groups were allowed to suckle and studied at the time of weaning (21 days old). At this time, body weight values remained higher in pups from MD rats than in controls, and although plasma glucose levels and liver TG concentration were also higher in pups from MD rats, plasma insulin levels did not differ between the 2 groups (Table 3).

Some pups from control and MD dams were weaned at the age of 21 days old, and studied at the age of 70 days. At this time, female rats coming from MD dams were still heavier (body weight 235.3 ± 3.3 g, n = 18) than those from controls (225.2 ± 3.6 g, n = 12, $P < .05$), whereas body weight in males did not differ (412.9 ± 7.4 g, n = 28, in those from MD dams and 427.7 ± 7.3 g, n = 19, in those from controls, nonsignificant [NS]). However, both females and males showed an altered glucose/insulin relationship. As shown in Figure 2, female pups coming from MD dams had an impaired OGTT as seen by the greater increase in plasma glucose, although plasma insulin levels did not differ as compared to the controls. As shown in Figure 3, male pups coming from MD dams show not only an impaired OGTT as a result of a greater increase in plasma glucose, but also a lower increase in plasma insulin, as compared to those coming from control dams.

In view of the differences found in the OGTTs, it was decided to separate values found in 70-day-old pups into those coming from MD dams that had more than 50% of the litter with macrosomia at delivery (estimated as pups whose birth weights were 1.7 SD greater than the mean birth weight of the control pups [35]) and those that had less than 50% of macrosomic pups. As shown in Figure 4, only female offspring from MD dams having litters with more than 50% macrosomic pups had impaired OGTT, whereas glucose tolerance did not differ in those from MD dams having litters with less than 50% macrosomic pups. In the case of males, as shown in Figure 5, the fact that they come from MD dams having either more or less than 50% macrosomic pups at the time of delivery did not modify the impaired glucose tolerance. Only those coming from dams having more than 50% of the pups macrosomic showed decreased plasma insulin as compared to the controls.

### DISCUSSION

In addition to showing the difficulties in obtaining a moderate and dose-dependent response to STZ treatment in rats, as seen by the normalization of the response in those rats receiving the 30-mg dose subsequent to an initial hyperglycemia, the present results also show that the degree of the response varies among rats after receiving the 35-mg/kg dose. Whether this different response was due to genetic variations of rats from the same strain remains to be established. Present findings also show that (i) neonatal macrosomia in offspring of MD rats is associated to neonatal hyperinsulinemia and mainly corresponds to an accumulation of body lipids; (ii) the enhanced body weight in the newborns from MD dams is maintained until the end of weaning, and in the case of female rats, until 70 days old; and (iii) a long-term altered OGTT is shown in the 70-day-old offspring from MD dams, which is specially manifested in those coming from dams having macrosomic litters rather than from normosomic, suggesting that macrosomia itself is an important

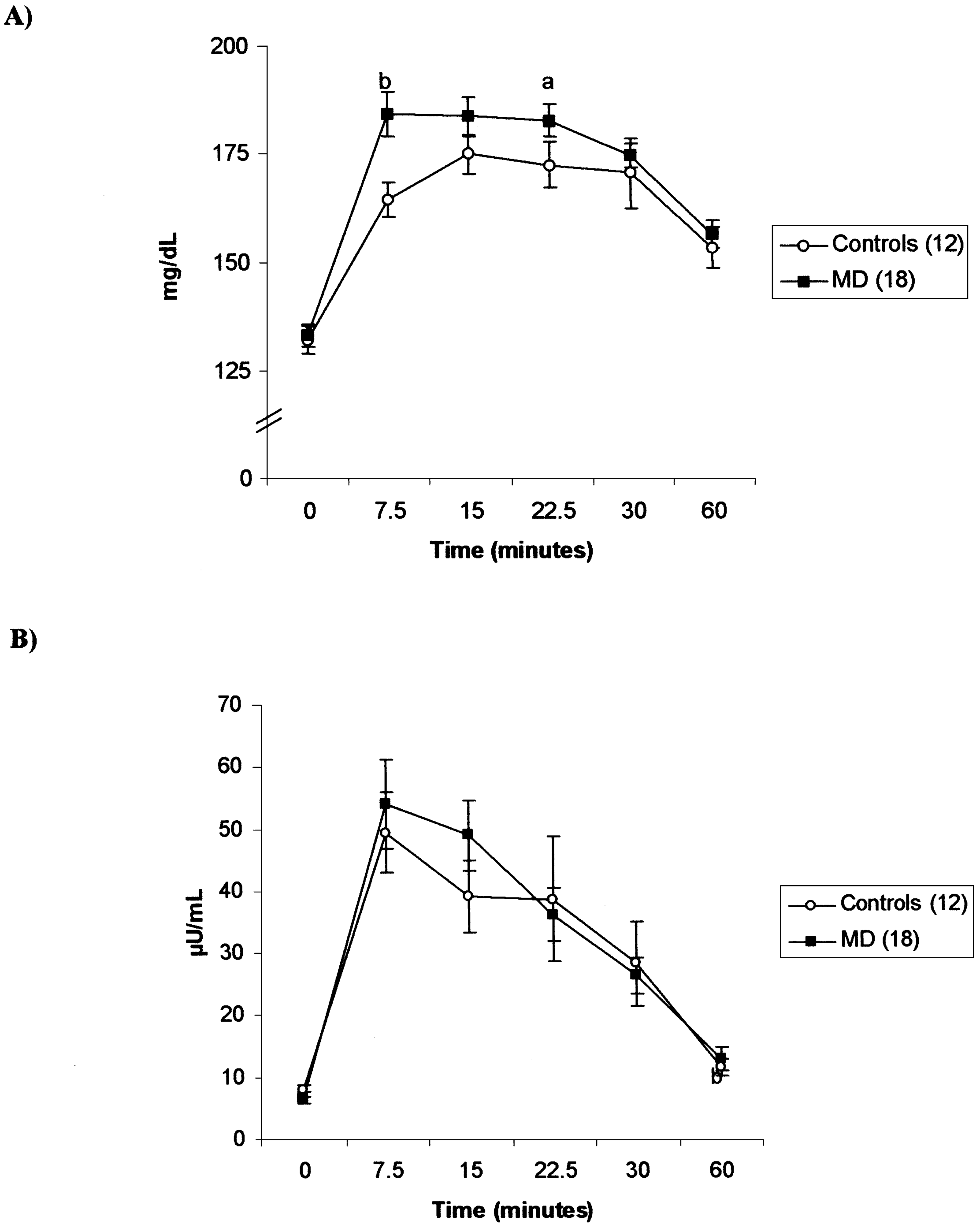

FIGURE 2
Plasma glucose (*A*) and insulin (*B*) levels at different times after an oral glucose load (2 g/kg body weight) at 70 days of age in female offspring of mildly streptozotocin diabetic (MD) and control rats. Significant differences versus controls at each time point are shown by the letters: a, $P < .05$; b, $P < .01$; c, $P < .001$. The number of rats per group are shown in parenthesis.

risk factor for long-term disturbance in the glucose/insulin relationship.

Different from what occurs in humans, macrosomia in the offspring of MD rats was found only after birth. This finding agrees with the fact that any previously reported effort to develop macrosomia in the offspring of STZ diabetic rats was obtained postnatally [26, 34–36, 44] and very rarely before birth, which agrees with the fact that in humans, fat depot

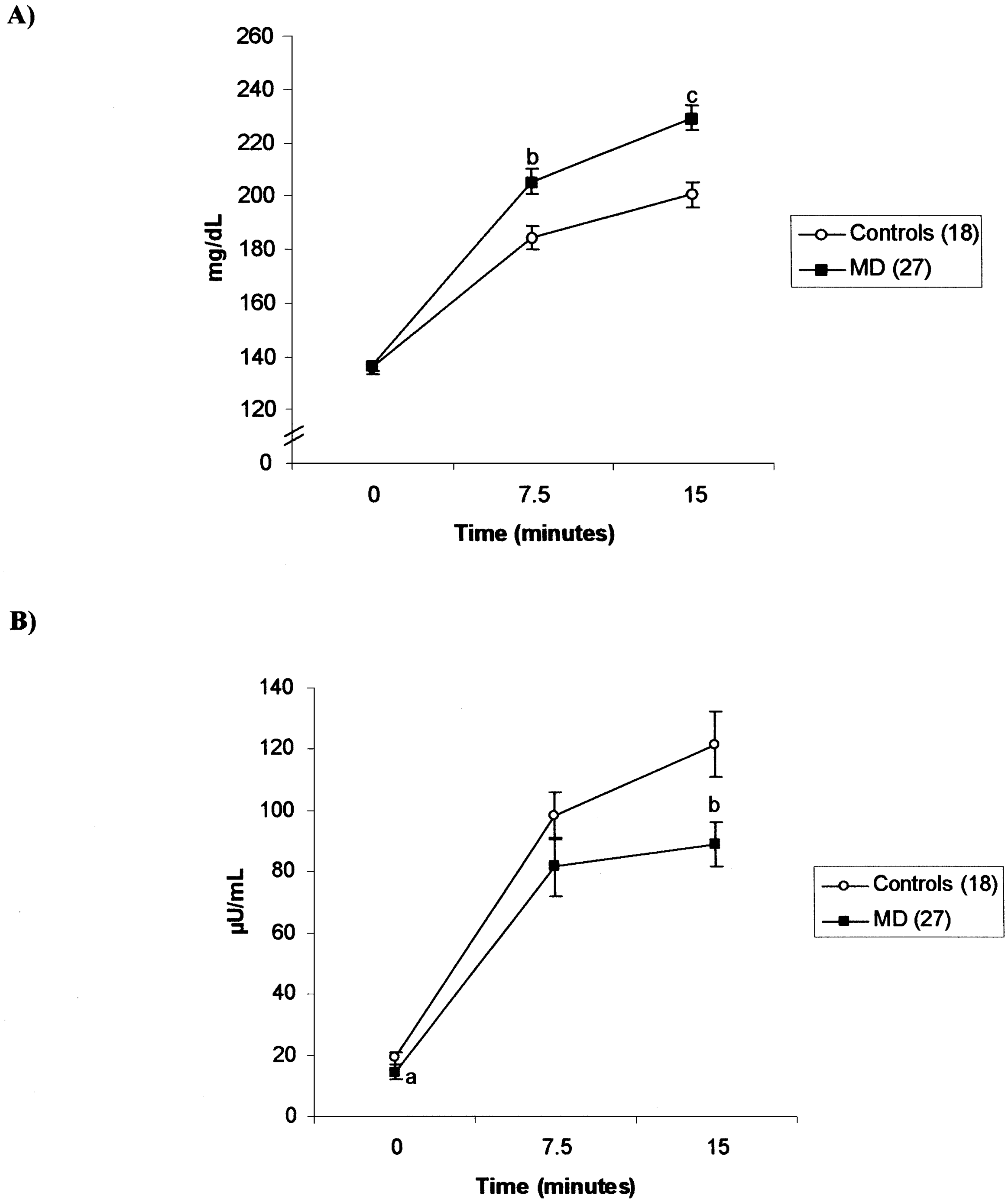

FIGURE 3

Plasma glucose (*A*) and insulin (*B*) levels at different times after an oral glucose load (2 g/kg body weight) at 70 days of age in male offspring of mildly streptozotocin diabetic (MD) and control rats. Significant differences versus controls at each time point are shown by the letters: a, $P < .05$; b, $P < .01$; c, $P < .001$. The number of rats per group are shown in parenthesis.

accumulation takes place intrauterinely, whereas in rats this occurs after birth (for a review, see [45]). An additional requirement for developing neonatal macrosomia in the mildly diabetic rat seems to be the presence of perinatal hyperinsulinemia, which must be a consequence of maternal hyperglycemia. Normoglycemia was, however, seen here in the MD rat at day 20 of pregnancy. Whereas the fetuses were hyperinsulinemic, a clear impaired maternal oral glucose tolerance was present

A)

B)

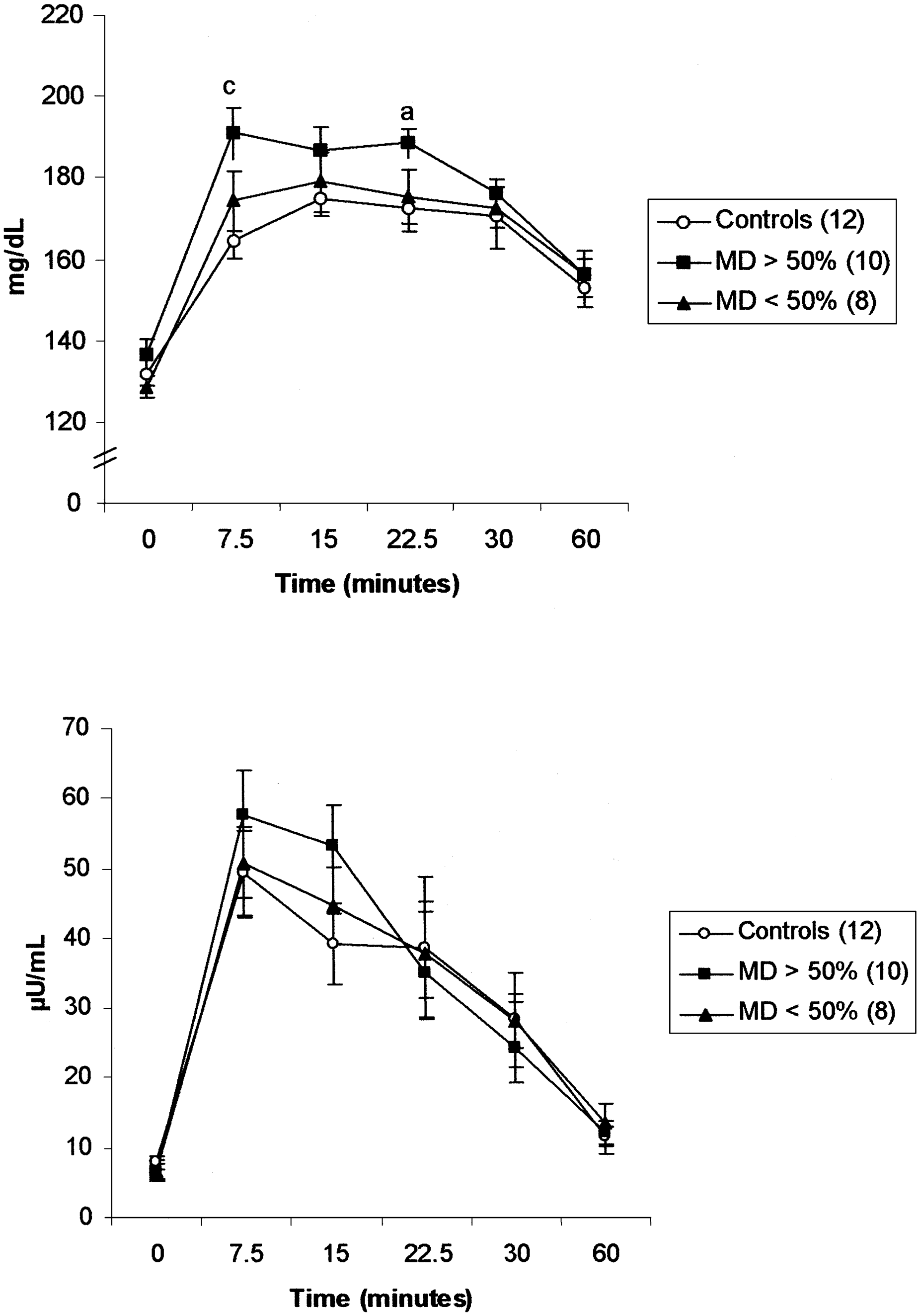

FIGURE 4

Plasma glucose (*A*) and insulin (*B*) levels at different times after an oral glucose load (2 g/kg body weight) at 70 days of age in female offspring of mildly streptozotocin diabetic (MD) rats that had either more or less than 50% of their litter macrosomic and control rats. Significant differences versus controls at each time point are shown by the letters: a, $P < .05$; b, $P < .01$; c, $P < .001$. The number of rats per group are shown in parenthesis.

in the dams. This indicates that whenever the mother eats, she develops hyperglycemia that will be followed by fetal hyperglycemic episodes and subsequent pancreatic stimulus. This action, plus the well-known enhanced pancreatic $\beta$-cell sensitivity to the glucose stimulus [21], justifies the hyperinsulinemia seen in the offspring of mildly diabetic rats around birth.

Enhanced body weight in the offspring of MD rats remained until weaning, and in the case of females, it was maintained

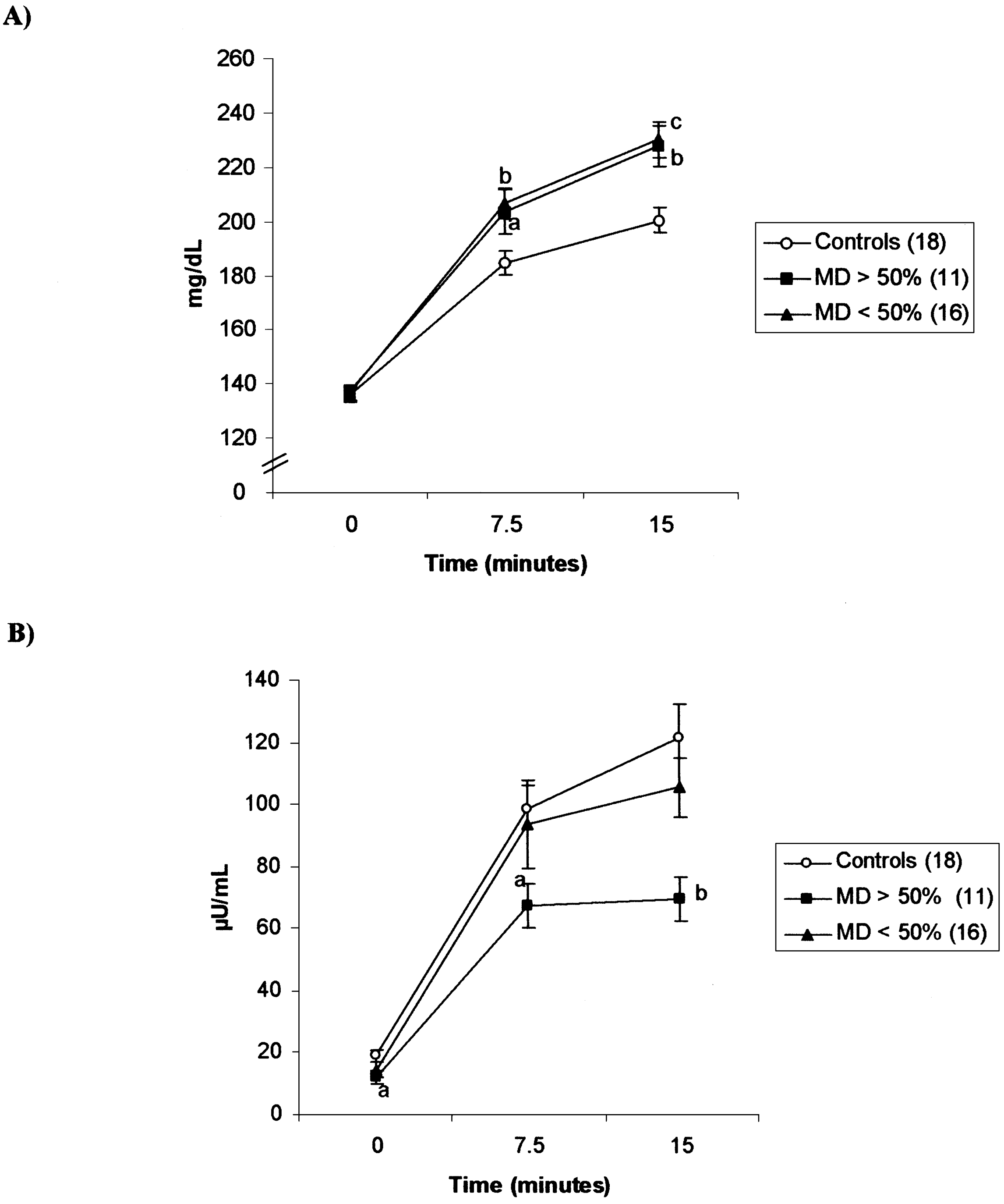

FIGURE 5
Plasma glucose ($A$) and insulin ($B$) levels at different times after an oral glucose load (2 g/kg body weight) at 70 days of age in male offspring of mildly streptozotocin diabetic (MD) rats that had either more or less than 50% of their litter macrosomic and control rats. Significant differences versus controls at each time point are shown by the letters: a, $P < .05$; b, $P < .01$; c, $P < .001$. The number of rats per group are shown in parenthesis.

until 70 days of age. This finding differs with previous reports where augmented body weight was maintained in both females and males until adulthood in macrosomic pups from mildly STZ diabetic rats [26, 34, 46]. However, in these studies, only pups showing macrosomia at birth were included in the study, and even on one occasion, nonmacrosomic pups born to STZ-treated dams were added to the control group [46]. All experimental designs allowed the respective authors to determine the consequences of postnatal macrosomia but had impeded determination of the specific long-term consequences of the diabetic intrauterine milieu. Because no pup selection was made at birth in the present study, it was possible here to clearly determine the long-term consequences of the intrauterine diabetic environment on the OGTT performed on the 70-day-old pups.

A long-term glucose/insulin alteration was seen in the offspring of the MD rats as shown by their impaired oral glucose tolerance at 70 days old. Although in the case of males, such alteration was consistently seen in all pups independent of their body weight at birth, in females, this was seen only in those coming from litters having more than 50% macrosomic pups. Small but significant differences in the OGTT were also previously found between macrosomic males and females coming from MD rat mothers as compared to those in controls [34, 46], although in these reports only macrosomic pups were included in the study. Thus, besides showing the long-term alteration in the glucose/insulin relationship in the offspring of MD rats, which confirms previous reports [21, 24, 30, 38, 47], the present study shows an impaired glucose tolerance in the presence of normal insulin levels in the adult female offspring of MD rats that had a high incidence of macrosomic pups but not in those having normosomic pups. The present findings, therefore, allow us to propose that macrosomia itself in the offspring of MD mothers is a risk factor for long-term disturbance of glucose/insulin relationships. Besides, these findings demonstrate the similarity to what happens in humans: the main component of macrosomia corresponds to an accumulation of fat depots, probably subsequent to perinatal hyperinsulinemia. It was also seen here that an enhanced liver TG concentration contributes to the total body fat accumulation in newborn pups from MD pups. This may also differ from humans, because lack of adipose tissue development in the newborn rat converts its liver into a temporal TG depot site, which is facilitated by the induction of lipoprotein lipase (LPL) activity taking place in the liver during the perinatal phase and the suckling period [45].

The condition of the SD pregnant rats, despite the moderate STZ dose administered, also deserves attention. These animals have normal basal insulin levels but greatly impaired insulinotropic response to the stimulus of the glucose load, indicating a major alteration in $\beta$-cell function, which is responsible for their hyperglycemia and exaggerated hypertriglyceridemia. Maternal hyperglycemia is also responsible for fetal hyperglycemia, which would have caused a prolonged fetal $\beta$-cell stimulus, resulting in depletion of fetal pancreatic insulin and subsequent hypoinsulinemia, contributing to the decrease in fetal body weight. Maternal hypertriglyceridemia may additionally be responsible for the increments in fetal plasma TG concentrations. Although TGs do not directly cross the placental barrier [48], the placenta has mechanisms by which maternal plasma TGs correlate with those in the fetus [49].

In summary, whereas the condition of those SD rats may be comparable to that found in uncontrolled pregnant diabetic women, where microsomia is most often found [50, 51], the MD rats mimic most of the changes taking place in gestational diabetic women. Thus, both diabetic populations obtained here with moderate amounts of STZ in rats may be used as appropriate experimental models to study the pathophysiological consequences of uncontrolled or moderately diabetic pregnant women, rather than the use of higher STZ doses that cause a diabetic condition in the rat that is quite different from the situation in pregnant women. Further, present findings show the long-term alteration of the glucose/insulin axis of the offspring of MD rats, magnified when they were macrosomic at birth.

### REFERENCES

[1] Martin, F. I., Heath, P., and Mountain, K. R. (1987) Pregnancy in women with diabetes mellitus. Fifteen years' experience. 1970–1985. *Med. J. Aust.*, **146,** 187–190.

[2] Fuhrmann, K., Reiher, H., Semmier, K., Fischer, F., Fischer, M., and Glockner, E. (1983) Prevention of congenital malformations in infants of insulin-dependent diabetic mothers. *Diabetes Care*, **6,** 219–223.

[3] Cowett, R. M., and Shwartz, R. (1982) The infant of the diabetic mother. *Pediatr. Clin. North Am.*, **29,** 1213–1231.

[4] Small, M., Cameron, A., Lunan, C. B., and MacCuish, A. C. (1987) Macrosomia in pregnancy complicated by insulin-dependent diabetes mellitus. *Diabetes Care*, **10,** 594–599.

[5] Pettitt, D. J., Aleck, K. A., Baird, H. R., Carraher, M. J., Bennett, P. H., and Knowler, W. C. (1988) Congenital susceptibility to NID-DM. Role of intrauterine environment. *Diabetes*, **37,** 622–628.

[6] Martin, A. O., Simpson, J. L., Ober, C., and Freinkel, N. (1985) Frequency of diabetes mellitus in mothers of probands with gestational diabetes: Possible maternal influence on the predisposition to gestational diabetes. *Am. J. Obstet. Gynecol.*, **151,** 471–475.

[7] Pettitt, D. J., Nelson, R. G., Saad, M. F., Bennett, P. H., and Knowler, W. C. (1993) Diabetes and obesity in the offspring of Pima Indian women with diabetes during pregnancy. *Diabetes Care*, **16,** 310–314.

[8] Pettitt, D. J., Baird, H. R., Aleck, K. H., Bennett, P. H., and Knowler, W. C. (1983) Excessive obesity in offspring of Pima Indian women with diabetes during pregnancy. *N. Engl. J. Med.*, **308,** 242–245.

[9] Vohr, B. B., Lipsitt, L. P., and Oh, W. (1980) Somatic growth of children of diabetic mothers and reference to birth size. *J. Pediatr.*, **97,** 196–199.

[10] Buchanan, T. A., Denno, K. M., Sipos, G. F., and Sadler, T. W. (1994) Diabetic teratogenesis: In vitro evidence for a multifactorial etiology with little contribution from glucose per se. *Diabetes*, **43,** 656–660.

[11] Ktorza, A., Nurjhan, N., Girard, J. R., and Picon, L. (1983) Hiperglycaemia induced by glucose infusion in the unrestrained pregnant rat: Effect on body weight and lipid synthesis in postmature fetuses. *Diabetologia*, **24,** 128–130.
[12] Gauguier, D., Bihoreau, M. T., Ktorza, A., Berthault, M. F., and Picon, L. (1990) Inheritance of diabetes mellitus as consequence of gestational hyperglycemia in rats. *Diabetes*, **39,** 734–739.
[13] Ward, D. T., Yau, S. K., Mee, A. P., Mawer, E. B., Miller, C. A., Garland, H. O., and Riccardi, D. (2001) Functional, molecular, and biochemical characterization of streptozotocin-induced diabetes. *J. Am. Soc. Nephrol.*, **12,** 779–790.
[14] Martin, M. E., Garcia, A. M., Blanco, L., Herrera, E., and Salinas, M. (1995) Effect of streptozotocin diabetes on polysomal aggregation and protein synthesis rate in the liver of pregnant rats and their offspring. *Biosci. Rep.*, **15,** 15–20.
[15] Martín, A., and Herrera, E. (1991) Different responses to maternal diabetes during the first and second half of gestation in the streptozotocin-treated rat. *Isr. J. Med. Sci.*, **27,** 442–448.
[16] Viana, M., Aruoma, O. I., Herrera, E., and Bonet, B. (2000) Oxidative damage in pregnant diabetic rats and their embryos. *Free Radic. Biol. Med.*, **29,** 1115–1121.
[17] Viana, M., Herrera, E., and Bonet, B. (1996) Teratogenic effects of diabetes mellitus in the rat. Prevention by vitamin E. *Diabetologia*, **39,** 1041–1046.
[18] Cederberg, J., Simán, C. M., and Eriksson, U. J. (2001) Combined treatment with vitamin E and vitamin C decreases oxidative stress and improves fetal outcome in experimental diabetic pregnancy. *Pediatr. Res.*, **49,** 755–762.
[19] Cederberg, J., and Eriksson, U. J. (2001) Increased rate of lipid peroxidation and protein carbonylation in experimental diabetic pregnancy. *Diabetologia*, **44,** 766–774.
[20] Simán, C. M., and Eriksson, U. J. (1997) Vitamin E decreases the occurrence of malformations in the offspring of diabetic rats. *Diabetes*, **46,** 1054–1061.
[21] Aerts, L., Holemans, K., and Van Assche, A. (1990) Maternal diabetes during pregnancy: Consequences for the offspring. *Diabet. Metab. Rev.*, **6,** 147–167.
[22] Aerts, L., and Van Assche, A. (1977) Rat fetal endocrine pancreas in experimental diabetes. *J. Endocrinol.*, **73,** 339–346.
[23] Aerts, L., and Van Assche, F. A. (1998) Ultrastructural evaluation of $\beta$-cell recruitment in virgin and pregnant offspring of diabetic mothers. *Diabetes Res. Clin. Pract.*, **41,** 9–14.
[24] Holemans, K., Aerts, L., and Van Assche, F. A. (1991) Evidence for an insulin resistance in the adult offspring of pregnant streptozotocin-diabetic rats. *Diabetologia*, **34,** 81–85.
[25] Holemans, K., Van Bree, R., Verhaeghe, J., Aerts, L., and Van Assche, F. A. (1993) In vivo glucose utilization by individual tissues in virgin and pregnant offspring of severely diabetic rats. *Diabetes*, **42,** 530–536.
[26] Merzouk, H., Madani, S., Sari, D. C., Prost, J., Bouchenak, M., and Belleville, J. (2000) Time course of changes in serum glucose, insulin, lipids and tissue lipase activities in macrosomic offspring of rats with streptozotocin-induced diabetes. *Clin. Sci.*, **98,** 21–30.
[27] Reynolds, W. A., Chez, R. A., Bhuyan, B. K., and Neil, G. L. (1974) Placental transfer of streptozotocin in the rhesus monkey. *Diabetes*, **23,** 777–782.
[28] Schein, P. S., and Loftus, S. (1968) Streptozotocin: Depression of mouse liver pyridine nucleotides. *Cancer Res.*, **28,** 1501–1506.
[29] Holemans, K., Van Bree, R., Verhaeghe, J., Meurrens, K., and Van Assche, F. A. (1997) Maternal semistarvation and streptozotocin-diabetes in rats have different effects on the in vivo glucose uptake by peripheral tissues in their female adult offspring. *J. Nutr.*, **127,** 1371–1376.
[30] Aerts, L., Holemans, K., and Van Assche, A. (1990) Impaired insulin response and action in offspring of severely diabetic rats. In: *Frontiers in Diabetes Research. Lessons from Animal Diabetes III*, edited by Shafrir, E., pp. 561–566. London, Smith-Gordon.
[31] Ishihara, G., Hiramatsu, Y., Masuyama, H., and Kudo, T. (2000) Streptozotocin-induced diabetic pregnant rats exhibit signs and symptoms mimicking preeclampsia. *Metabolism*, **49,** 853–857.
[32] Gerber, R. T., Holemans, K., O'Brien-Coker, I., Mallet, A. I., Van Bree, R., Van Assche, F. A., and Poston, L. (2000) Increase of the isoprostane 8-isoprostaglandin $F_{2\alpha}$ in maternal and fetal blood of rats with streptozotocin-induced diabetes: Evidence of lipid peroxidation. *Am. J. Obstet. Gynecol.*, **183,** 1035–1040.
[33] Plagemann, A., Harder, T., Rake, A., Melchior, K., Rittel, F., Rohde, W., and Dörner, G. (1998) Hypothalamic insulin and neuropeptide Y in the offspring of gestational diabetic mother rats. *Neuroreport*, **9,** 4069–4073.
[34] Oh, W., Gelardi, N. L., and Cha, C. M. (1991) The cross-generation effect of neonatal macrosomia in rat pups of streptozotocin induced diabetes. *Pediatr. Res.*, **29,** 606–610.
[35] Gelardi, N. L., Cha, C. M., and Oh, W. (1990) Glucose metabolism in adipocytes of obese offspring of mild hyperglycemic rats. *Pediatr. Res.*, **28,** 641–645.
[36] Mulay, S., Philip, A., and Solomon, S. (1983) Influence of maternal diabetes on fetal rat development: Alteration of insulin receptors in fetal liver and lung. *J. Endocrinol.*, **98,** 401–410.
[37] Ryan, E. A., Tobin, B. W., Tang, J., and Finegood, D. T. (1993) A new model for the study of mild diabetes during pregnancy: Syngeneic islet-transplanted STZ-induced diabetic rats. *Diabetes*, **42,** 316–323.
[38] Ryan, E. A., Liu, D. T., Bell, R. C., Finegood, D. T., and Crawford, J. (1995) Long term consequences in offspring of diabetes in pregnancy: Studies with syngeneic islet-transplanted streptozotocin-diabetic rats. *Endocrinology*, **136,** 5587–5592.
[39] Eriksson, U., Dahlstrom, E., Larsson, K. S., and Hellerstrom, C. (1982) Increased incidence of congenital malformations in the offspring of diabetic rats and their prevention by maternal insulin. *Diabetes*, **31,** 1–6.
[40] Boloker, J., Gertz, S. J., and Simmons, R. A. (2002) Gestational diabetes leads to the development of diabetes in adulthood in the rat. *Diabetes*, **51,** 1499–1506.
[41] Munilla, M. A., and Herrera, E. (1997) A cholesterol-rich diet causes a greater hypercholesterolemic response in pregnant than in nonpregnant rats and does not modify fetal lipoprotein profile. *J. Nutr.*, **127,** 2239–2245.
[42] Folch, J., Lees, M., and Sloane Stanley, G. H. (1957) A simple method for the isolation and purification of total lipids from animal tissues. *J. Biol. Chem.*, **22,** 24–36.
[43] Ruiz, J. I., and Ochoa, B. (1997) Quantification in the subnanomolar range of phospholipids and neutral lipids by monodimension thin-layer chromatography and image analysis. *J. Lipid Res.*, **38,** 1482–1489.
[44] Aerts, L., and Van Assche, A. (1981) Endocrine pancreas in the offspring of rats with experimentally induced diabetes. *J. Endocrinol.*, **88,** 81–88.
[45] Herrera, E., and Amusquivar, E. (2000) Lipid metabolism in the fetus and the newborn. *Diabetes Metab. Res. Rev.*, **16,** 202–210.

[46] Gelardi, N. L., Cha, C. M., and Oh, W. (1991) Evaluation of insulin sensitivity in obese offspring of diabetic rats by hyperinsulinemic-euglycemic clamp technique. *Pediatr. Res.*, **30**, 40–44.

[47] Plagemann, A., Harder, T., Melchior, K., Rake, A., Rohde, W., and Dörner, G. (1999) Elevation of hypothalamic neuropeptide Y-neurons in adult offspring of diabetic mother rats. *Neuroreport*, **10,** 3211–3216.

[48] Herrera, E., Bonet, B., and Lasunción, M. A. (1998) Maternal-fetal transfer of lipid metabolites. In: *Fetal and Neonatal Physiology*, edited by Polin, R. A., and Fox, W. W., pp. 447–458. Philadelphia, W. B. Saunders.

[49] Herrera, E. (2002) Implications of dietary fatty acids during pregnancy on placental, fetal and postnatal development—A review. *Placenta*, **23,** S9–S19.

[50] Casson, I. F., Clarke, C. A., Howard, C. V., McKendrick, O., Pennycook, S., Pharoah, P. O. D., Platt, M. J., Stanisstreet, M., Van Velszen, D., and Walkinshaw, S. (1997) Outcomes of pregnancy in insulin dependent diabetic women: Results of a five year population cohort study. *BMJ*, **315,** 275–278.

[51] Ekbom, P., Damm, P., Feldt-Rasmussen, B., Feldt-Rasmussen, U., Molvig, J., and Mathiesen, E. R. (2001) Pregnancy outcome in type 1 diabetic women with microalbuminuria. *Diabetes Care*, **24,** 1739–1744.

